# Oligodendroglial tumours: subventricular zone involvement and seizure history are associated with CIC mutation status

**DOI:** 10.1186/s12883-019-1362-y

**Published:** 2019-06-18

**Authors:** Zhenyin Liu, Hongsheng Liu, Zhenqing Liu, Jing Zhang

**Affiliations:** Department of medical imaging, Guangzhou women and children’s medical center, Guangzhou medical university, Jinsui road 9 #, Guangzhou City, 510623 People’s Republic of China

**Keywords:** Oligodendroglial Tumours, CIC mutation, Subventricular zone involvement, Logistic regression, Seizure history

## Abstract

**Background:**

CIC-mutant oligodendroglial tumours linked to better prognosis. We aim to investigate associations between CIC gene mutation status, MR characteristics and clinical features.

**Methods:**

Imaging and genomic data from the Cancer Genome Atlas and the Cancer Imaging Archive (TCGA/TCIA) for 59 patients with oligodendroglial tumours were used. Differences between CIC mutation and CIC wild-type were tested using Chi-square test and binary logistic regression analysis.

**Results:**

In univariate analysis, the clinical variables and MR features, which consisted 3 selected features (subventricular zone[SVZ] involvement, volume and seizure history) were associated with CIC mutation status (all *p* < 0.05). A multivariate logistic regression analysis identified that seizure history (no vs. yes odd ratio [OR]: 28.960, 95 confidence interval [CI]:2.625–319.49, *p* = 0.006) and SVZ involvement (SVZ- vs. SVZ+ OR: 77.092, *p* = 0.003; 95% CI: 4.578–1298.334) were associated with a higher incidence of CIC mutation status. The nomogram showed good discrimination, with a C-index of 0.906 (95% CI: 0.812–1.000) and was well calibrated. SVZ- group has increased (SVZ- vs. SVZ+, hazard ratio [HR]: 4.500, *p* = 0.04; 95% CI: 1.069–18.945) overall survival.

**Conclusions:**

Absence of seizure history and SVZ involvement (−) was associated with a higher incidence of CIC mutation.

**Electronic supplementary material:**

The online version of this article (10.1186/s12883-019-1362-y) contains supplementary material, which is available to authorized users.

## Background

Low-grade gliomas (LGGs) exhibiting oligodendroglial features include oligodendrogliomas and oligoastrocytomas [1]. The updated 2016 edition of the World Health Organization (WHO) Classification of tumors of the Central Nervous System (CNS) uses molecular parameters and the histology to define the main tumor categories for the first time. This represents a shift from the traditional principle of using neuropathological diagnoses, which are primarily based on the microscopic features, to using molecularly-oriented diagnoses [2, 3]. Therefore, neurosurgeons increasingly depend on molecular genetic features to guide their clinical judgement and decision-making processes [4, 5]. LGG samples with an isocitrate dehydrogenase (IDH) mutation and the codeletion of 1p and 19q had the most favourable outcomes for treatment [6, 7].

The Capicua transcriptional repressor (CIC) gene is often mutated in oligodendroglial tumours with the codeletion of 1p and 19q. CIC-mutant oligodendroglial tumours are also associated with better prognoses [8, 9] and to better treatment outcomes and overall survival [8–10]. In the field of MR radiogenomics research, many imaging-based characteristics (tumour localization, mass effect, tumour contrast enhancement, etc.) are correlated with molecular genetic biomarkers (EGFR, IDH/1p19q subtype, TP53 mutation status, etc.) that are used to identify their phenotypes upon imaging [8, 11–15]. Recent studies have also found that the heterogeneity in patient prognoses might be linked to neuronal stem cells (NSCs), located in the subventricular zone (SVZ) [8, 16, 17].

To date, although several studies have evaluated MRI characteristics as they relate to IDH/1p19q status [18, 19], no study has investigated associations between CIC mutation status and MR imaging features in oligodendroglial tumours. Radiological detection of CIC mutation status may facilitate the preoperative prediction of a patient’s prognosis. Therefore, this paper reports preliminary research that can be used to determine the associations between CIC gene mutation status, MR characteristics and clinical features.

## Methods

### Patient population

All patient data was acquired from the published The Cancer Genome Atlas LowGrade Glioma (TCGA-LGG) project and within this publication it is stated “Specimens were obtained from patients, with appropriate consent from institutional review boards”. (http://cancergenome.nih.gov/).

The clinical files of oligodendroglial tumours were downloaded from the TCGA data portal (https://tcgadata.nci.nih.gov/tcga/dataAccessMatrix.htm;updated: 2018-08-23). MR datawere provided by TCIA (updated: 2014-09-04).TCGA and TCIA are publicly available databases that contain no linkage to patient identifiers. All patients must meet the following criteria to enter our study: available pathologic diagnosis of oligodendroglial tumours (oligodendroglioma or oligoastrocytoma) from TCGA; available CIC mutation status (CIC mutation or CIC wild-type) from TCGA [20] (extracted from a previous study [N Engl J Med. 2015; 372 (26):2481–2498]); and available MR images (T1WI, T2WI, Flair and post-contrast) from TCIA. Finally, 59 patients with oligodendroglial tumours were used in this institutional review board approval–exempt study.

### Image feature analysis

The MR images were presented to two radiologists for interpretation and measurement and in cases of disagreement a consensus was reached after discussion. Both radiologists were blind to CIC mutation status and clinical information. The following 7 qualitative MR imaging features [11, 12, 21, 22] were evaluated: (1) volume (< 60 cm^3^ vs. ≥60 cm^3^); (2) multifocal (no vs. yes); (3) intratumoural haemorrhaging (no vs. yes); (4) enhancing margin (well defined vs. poorly defined); (5) necrosis (no vs. yes); (6) proportion of contrast-enhanced tumour (< 5% vs. ≥5%); and (7) SVZ Involvement (no vs. yes). The tumours that were located in close contact with the SVZ were classified as SVZ+, while the tumours that were located distantly from the SVZ were classified as SVZ-^17^.

### Statistical analysis

Differences between CIC mutation and CIC wild-type were tested using the Chi-square test and binary logistic regression analysis (version 22.0; SPSS Company, Chicago, IL). Odd ratios (OR) and 95% confidence intervals (CI) are reported. The area under the receiver operator characteristic curve (AUC) was estimated for prediction of CIC gene mutation status. The sensitivity, specificity, positive likelihood ratio (PLR) and negative likelihood ratio (NLR) of the model in the prediction of CIC mutations were obtained. Survival analysis (SVZ- vs. SVZ+) was estimated using the Cox proportional hazards models. Hazard ratios (HR) and 95% CI are reported. The statistical significance threshold was set at a *P*-value of 0.05 (two-sided) to indicate statistical significance.

## Results

**Fig. 1:**
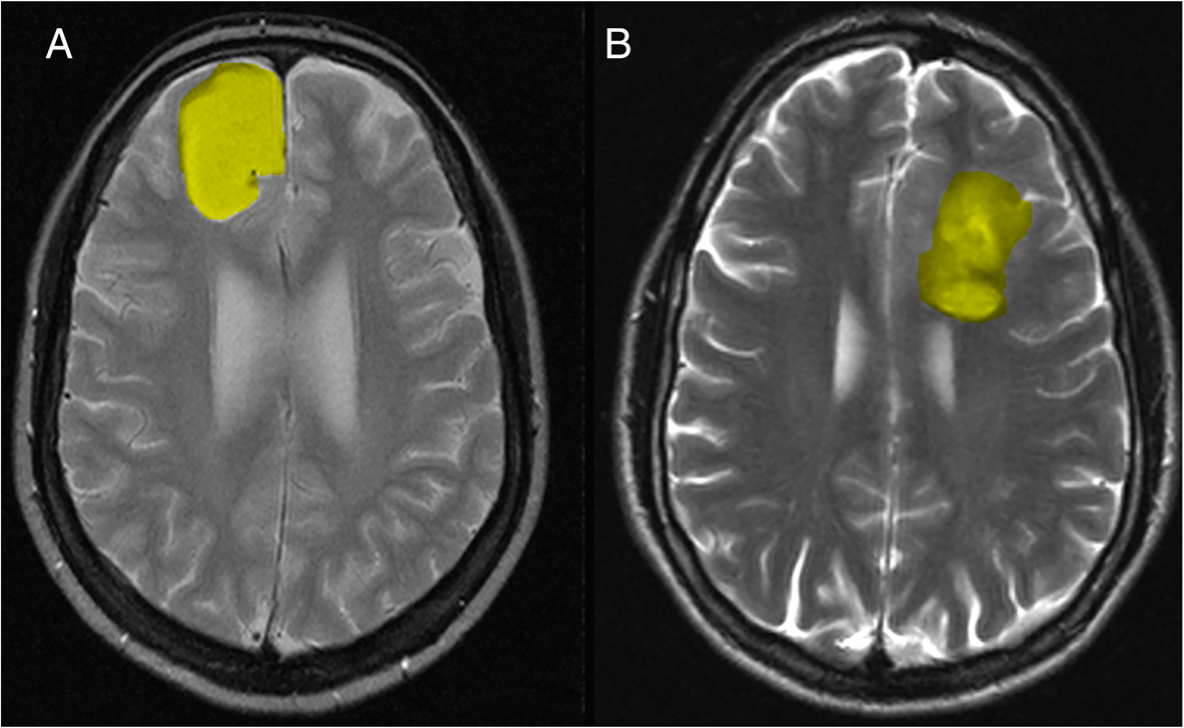
Tumor (T2-weighted MR images) localization in the cortex without subventricular zone involvement (**a**). Tumor (T2-weighted MR images) with infiltration of the subventricular zone (**b**)

Our series of 59 patients (45.4 ± 14.0 years, range: 18–74 years) included 29 men (49.2%) and 30 (50.8%) women. CIC mutations were found in 10 (16.9%) of 59 patients. There were 35 patients (59.3%) with tumours that involved the SVZ (Fig. 1). Specifically, 8 tumours involved the frontal horn, 5 the body, 1 the occipital horn, 17 the temporal horn and 4 others. The cohort mean OS was 37.5 months (range: 0.03–156.11 and median = 21.4), and 44 patients were still alive (74.6%) at the end of the study while 15 were deceased (25.4%). Clinical data are summarized in Table 1 and Additional file 1: Table S1.

**Table 1:** Association between clinical and MR features and CIC mutation status (follow up: 0.03–156.11 months)

	No. of CIC Wild-Type	No. of CIC Mutations	χ^2^	*P*
Age			0.08	0.777
< 40 year	20	3		
> =40 years	29	7		
Sex			0.083	0.773
Female	24	6		
Male	25	4		
Race Category			0.179	0.672
Black or African American	3	1		
White	46	9		
Seizure History			7.538	0.023*
No	12	7		
Yes	36	3		
KPS			3.334	0.068
< 80	4	2		
> =80	27	2		
Primary Tumor Laterality			0.183	0.669
Left	18	5		
Right	31	5		
Volume			7.687	0.006*
< 60 cm^3^	10	7		
> =60 cm^3^	39	3		
Multifocal			0.234	0.651
No	48	10		
Yes	1	0		
Intratumoral hemorrhage			0.308	0.579
No	45	8		
Yes	4	2		
Enhancing margin			1.632	0.201
Well Defined	18	1		
Poorly Defined	31	9		
Necrosis			< 0.001	1
No	6	1		
Yes	43	9		
Proportion contrast-enhanced tumor			2.649	0.104
< 5%	34	10		
> =5%	15	0		
Subventricular Zone Involvement			9.802	0.002*
No	15	9		
Yes	34	1		

*P < 0.05

**Fig. 2:**
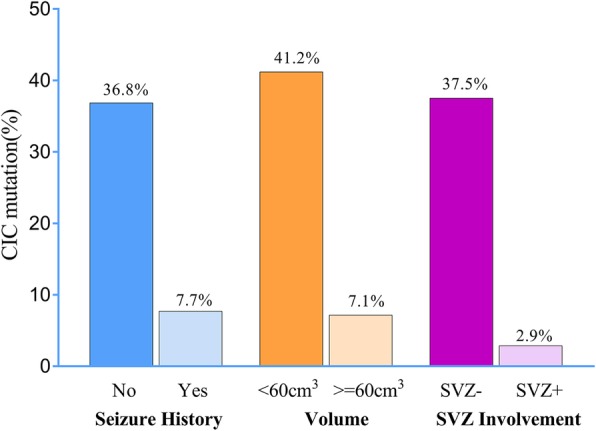
Smaller tumor volume, SVZ- and absent seizure history were associated with a significantly higher incidence of CIC mutation (univariate analysis *P* < 0.05)

In univariate analysis, the clinical variables and MR features, which consisted of three selected features (SVZ involvement, volume and seizure history) were significantly associated with CIC mutation status (all *P* < 0.05). We demonstrated that a smaller tumour volume (OR: 9.100, *P* = 0.004), SVZ-(OR: 20.400 *P* = 0.006) and a history absent of seizures (OR: 6.462, *P* = 0.014) were associated with a significantly higher incidence of CIC mutations (Table 1 Fig. 2).

**Fig. 3:**
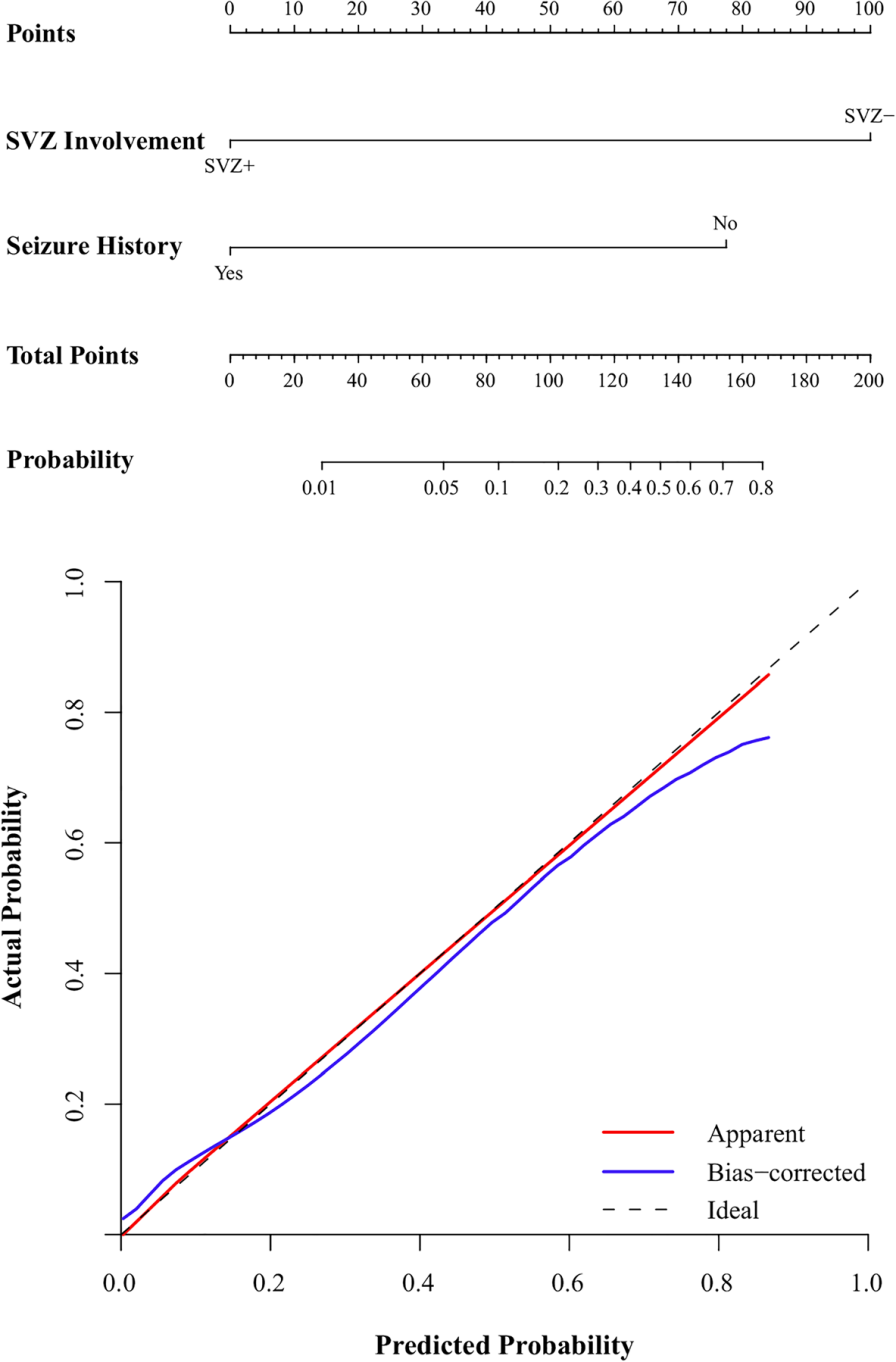
Multivariate analysis showed that absence of seizure history and SVZ involvement (−) was associated with a higher incidence of CIC mutation. The nomogram showed good discrimination, with a C-index of 0.906 (95% CI: 0.812–1.000) and was well calibrated

**Fig. 4:**
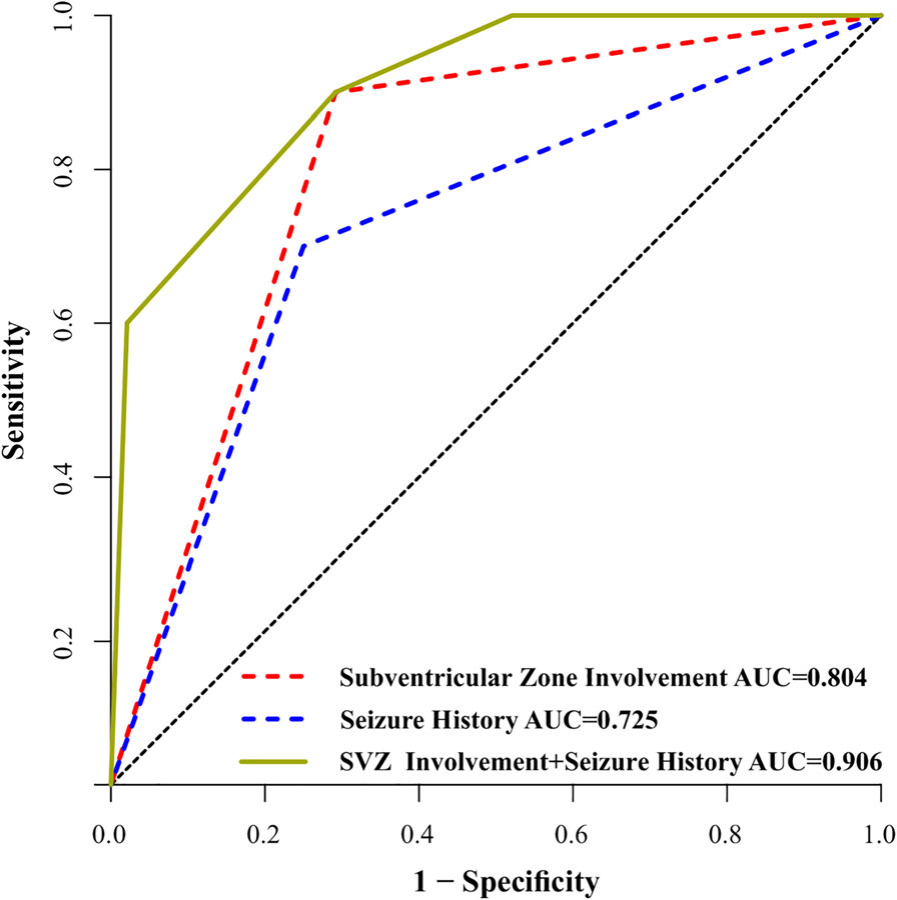
ROC curve analysis showed that subventricular zone involvement of oligodendroglial tumors in combination with seizure history may therefore be used to prognosticate CIC mutation status better than use of variables alone

In multivariate logistic regression analysis, only two risk factors were significant independent predictors (Table 2). We demonstrated that seizure history (no vs. yes OR: 28.960, 95CI:2.625–319.49, *P* = 0.006) and subventricular zone involvement (SVZ- vs. SVZ+ OR: 77.092, *P* = 0.003; 95% CI: 4.578–1298.334) were associated with a higher incidence of CIC mutation status. The nomogram displayed high discrimination, with a C-index of 0.906 (95% CI: 0.812–1.000) and was well calibrated (Fig. 3). The sensitivity, specificity, positive likelihood ratio (PLR) and negative likelihood ratio (NLR) of this model in the prediction of CIC mutations were 0.90, 0.71, 3.09 and 0.14, respectively. Subventricular zone involvement (−) of oligodendroglial tumours in combination with absence of seizure history may therefore be used to better prognosticate CIC mutation status than the use of each variable alone (Fig. 4).

**Table 2:** binary logistic regression analysis of prognostic factors for CIC mutation status

	Wald	Sig.	OR	95.0% CI for OR	
				Lower	Upper
Subventricular Zone Involvement	9.095	0.003*	77.092	4.578	1298.334
Seizure History	7.551	0.006*	28.960	2.625	319.49
Constant	14.378	0.000*	0.003		

*P < 0.05

**Fig. 5:**
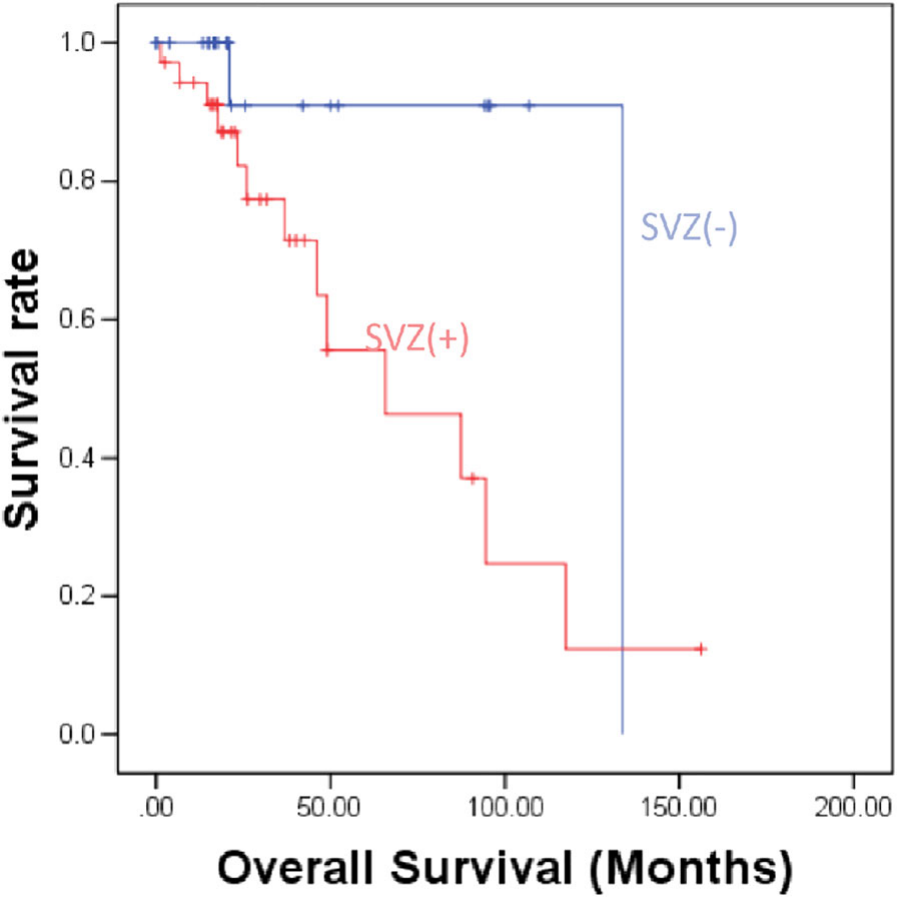
Overall survival outcomes of patients with tumors contacting the SVZ, and those with tumors not involved in the SVZ (log rank = 5.029, *P* = 0.025)

Patients (follow up: 0.03–156.11 months) with SVZ- had a longer median overall survival (133.6 vs.65.7) months (SVZ- vs. SVZ+, hazard ratio (HR): 4.500, *P* = 0.04; 95% CI: 1.069–18.945) than patients with SVZ+(Fig. 5). SVZ+ tumors were significantly larger than SVZ- tumors (181 ± 92.1 cm^3^ vs.117 ± 116 cm^3^, *P* < 0.05).

## Discussion

Molecular genetic studies demonstrated distinct glioma entities with specific epigenetic and genetic profiles [23]. Some oligoastrocytomas and most oligodendrogliomas are characterized by a typical and unique unbalanced translocation, der (1, 19), resulting in a 1p/19q co-deletion (codeletion of 1p and 19q). Candidate tumour suppressor genes (TSGs) targeted by these losses, including FUBP1 on 1p31.1 and CIC on 19q13.2, were only recently discovered [10]. CIC-mutant oligodendroglial tumours are also linked to better prognoses [8, 9].

There are a number of studies regarding the relationship between imaging features and gene mutations. Rios Velazquez E et al. [24] confirmed that quantitative features related to intratumour heterogeneity that were able to successfully discriminate (AUC = 0.69) between EGFR- and EGFR+ lesions. Brendle C et al. [25] showed that the differentiation of high-grade gliomas and low-grade gliomas (sensitivity: 100% specificity: 80%) is made possible by the dynamic contrast-enhanced MR perfusion parameter Ve (*P* = 0.024), while arterial spin labelling perfusion shows the potential for the discrimination of the ATRX and IDH mutation statuses (sensitivity: 75% specificity: 88%, *P* = 0.014). Dagher J [26] implied that wild-type von Hippel-Lindau (VHL) renal cell carcinomas were associated with lymph nodal metastases. Rizzo S [27] found that a pleural effusion related to ALK mutations while nodules located in non-tumour lobes or round lesion shapes were related to a KRAS mutation in subgroups of non-small cell lung cancer patients. So far, no study has investigated associations between CIC mutation status and MR imaging features in oligodendroglial tumours. This study suggests that SVZ involvement and seizure history can be conveniently used to facilitate the prediction of CIC mutation status.

The subventricular zone has been associated with the origination and development, as well as the biological behaviour of LGGs [22]. Recently, limited studies have reported associations between SVZ involvement and patient prognosis. Nakagawa Y et al. [28] implied that the loss of 19q and lack of SVZ+ might be prognostic for longer survival. Liu S et al. [22] reported that multivariate analysis showed that a shorter distance between the tumor centroid and the SVZ (*p* = 0.039) was significantly associated with poor overall survival in SVZ-involved patients (low-grade astrocytoma). Liu S et al. [22] also reported that a longer distance between the SVZ and the tumour centroid was significantly related to better overall survival in SVZ-involved LGG patients. Adeberg S et al. [8] confirmed that the tumour location with regard to the subventricular zone is related to a patient’s prognosis (*p* < 0.05).

Nomograms are user-friendly tools that give relative contexts and probabilities of cancer prognoses [11]. In the present study, we constructed three models for the preoperative prediction of CIC mutation statuses in oligodendroglial tumours. ROC curve analysis showed that use of seizure history combined with SVZ involvement (AUC = 0.906) was superior to simply the use of seizure history (AUC = 0.725) or SVZ involvement alone (AUC = 0.804). The discriminatory power of the nomogram which combined SVZ involvement and seizure history was also very strong and well calibrated.

The limitations of our study included the retrospective nature of our data collection, the relatively limited number of cases (*n* = 59) and the automatic imaging feature extraction not being implemented. In addition, we do not know whether re-classification by an expert neuropathologist has been performed, however, similar to other papers (2017–2019 more than 250 articles) published using this dataset, we therefore believe our conclusions are not largely influence by such factors. Further study should be conducted by using a larger pool of oligodendroglial tumours patients with the use quantitative image analysis tools being required [29]. The newly emerged field of radiogenomics allows specific MR imaging phenotypes to be linked with gene expression profiles. Further work is needed to better define the relationships identified in our study and to explore additional relationships (SVZ involvement and CD133, SVZ involvement and CD44, SVZ involvement and MMPs).

## Conclusions

In conclusion, this study presents that SVZ involvement (−) and absence of seizure history may therefore be used to facilitate the prediction of CIC mutation status. Patients with SVZ- had a longer median overall survival months than patients with SVZ+.This work represents a practical application of imaging findings for personalized medicine. External validation of this model in other cohorts of patients is needed.

## Additional file

Additional file 1: **Table S1.** Clinical data of this study. (XLS 71 kb)

## Data Availability

All data generated or analyzed in this study are included in this published article and its Additional files.

## Abbreviations

AUC: Area under the curve
CI: Confidence interval
CIC: Capicua transcriptional repressor
HR: Hazard ratio
IDH: Isocitrate dehydrogenase
LGG: Low-grade gliomas
NLR: Negative likelihood ratio
NSCs: Neuronal stem cells
OR: Odds ratio
PLR: Positive likelihood ratio
SVZ: Subventricular zone
TCIA: The Cancer Imaging Archive
TSGs: Tumor suppressor genes
